# Failure patterns and survival outcomes in triple negative breast cancer (TNBC): a 15 year comparison of 448 non-Hispanic black and white women

**DOI:** 10.1186/s40064-016-2444-6

**Published:** 2016-06-17

**Authors:** Shreya Prasad, Jimmy T. Efird, Sarah E. James, Paul R. Walker, Timothy M. Zagar, Tithi Biswas

**Affiliations:** Department of Internal Medicine, North Shore-Long Island Jewish Medical Center, Manhasset, NY USA; Center for Health Disparities, Brody School of Medicine, Office of Research, College of Nursing, East Carolina University, Greenville, NC USA; Department of Radiation Oncology, Mayo Clinic, Rochester, MN USA; Division of Hematology/Oncology, Department of Internal Medicine, East Carolina University, Greenville, NC USA; Department of Radiation Oncology and Neurosurgery, Cyberknife Radiosurgery Program, University of North Carolina at Chapel Hill, Chapel Hill, NC USA; Department of Radiation Oncology, University Hospitals Sideman Cancer Center, Case Western Reserve University, Cleveland, OH USA

## Abstract

**Purpose:**

Triple negative breast cancer (TNBC) is a distinct subtype of breast cancer with unique pathologic, molecular and clinical behavior. It occurs more frequently in young blacks and has been reported to have a shorter disease-free interval. We undertook this study to analyze the demographic characteristics, failure patterns, and survival outcomes in this disease.

**Methods:**

A total of 448 non-Hispanic black and white women were identified over a 15 year period from 1996 to 2011. Demographic and clinical information including age, race, menopausal status, stage, tumor characteristics, and treatments were collected. Fisher’s exact test and multivariable Cox regression were used to compare failure patterns and survival outcomes between races.

**Results:**

49 % (n = 223) were black. 59 % patients were between 41 and 60 years, with 18 % ≤40 years. 57 % were premenopausal and 89 % had grade 3 tumors. Stage II (47 %) was most frequent stage at diagnosis followed by stage III (28 %). 32 % had lymphovascular invasion. Adjusting for age, stage, and grade, there was no difference in survival outcomes (OS, DFS, LFFS, and DFFS) between the two races. 62 (14 %) patients failed locally either in ipsilateral breast or chest wall, and 19 (4 %) failed in the regional lymphatics. Lung (18 %) was the most frequent distant failure site with <12 % each failing in brain, liver and bones.

**Conclusion:**

Failure patterns and survival outcomes did not differ by race in this large collection of TNBC cases. Lung was the predominate site of distant failure followed by brain, bone, and liver. Few patients failed in the regional lymphatics.

## Background

Breast cancer is the most common malignancy among women in developed countries.

Triple-negative breast cancer (TBNC) is a distinct subtype of breast cancer with unique pathological, molecular and clinical behavior. Phenotypically, these tumors are negative for estrogen-receptor (ER), progesterone-receptor (PR) and human epidermal growth factor receptor 2 (HER2). TNBC often is used synonymously with basal subtype breast cancer, which is one of 5 subtypes based on gene expression obtained from microarray data. However, there exists 20–30 % discordance between these two entities (Anders and Carey 2009). TNBC accounts for approximately 12–17 % of all breast cancers (Anders and Carey 2009; Foulkes et al. 2010), corresponding to over 172,000 patients among an estimated 1 million cases of breast cancer diagnosed annually worldwide (Anders and Carey 2009). Owing to lack of receptor expression, TNBCs are not candidates for targeted biological therapies.

TNBCs are reported to have an aggressive clinical course, worse prognosis, and a shorter disease-free interval and overall survival compared with receptor positive breast cancers (Anders and Carey 2009; Rhee et al. 2008; Tian et al. 2008; Nishimura and Arima 2008). Although these tumors tend to recur earlier than luminal breast cancers in the first 5 years following diagnosis, this aggressiveness appears to diminish beyond this timeframe (Dent et al. 2007). The visceral organs and soft tissue are common sites of recurrence, with lower rates of bone involvement (Liedtke et al. 2008).

Pre-menopausal black women have a higher incidence of TNBC than white women, which suggests possible differences on a molecular level (Carey et al. 2006; Bauer et al. 2007a; Morris et al. 2007). However, the exact causal mechanism is not fully understood. While several studies have focused on disparate outcomes for TNBC among black women, findings have been inconsistent (National Cancer Institute 1990; Bradley et al. 2002; Ayanian et al. 1993; McWhorter and Mayer 1987; Furberg et al. 2001; Ihemelandu et al. 2007).

We undertook this study to compare failure patterns and survival outcomes between non-Hispanic white and black women diagnosed with TNBC.

## Methods

Patients for this study were collected from two large, tertiary referral centers in the southeastern region of the United States. Women with triple negative breast cancer (TNBC), defined as negative estrogen, progesterone and HER2 receptor status (<1 % of tumor cells demonstrating positive nuclear staining on immunohistochemistry), were identified from the institution tumor registry after obtaining the Institutional Review Board approval. Between 1996 and 2011, a total of 448 non-Hispanic women were identified and are included in this analysis. The medical records of each patient were reviewed to extract information. We collected information on both demographic and tumor characteristics including age, race, menopausal status, insurance type, pathology, grade, stage and lymphovascular invasion (LVI). All patients were staged based on AJCC—6th edition after reviewing both clinical and radiographical information. Other pertinent information collected were type of treatments received including surgery, chemotherapy and radiation therapy. Surgery included partial mastectomy, mastectomy or none. Chemotherapy was categorized by neoadjuvant and adjuvant chemotherapy or none. Radiation was categorized as yes or no.

We collected detail information on tumor failure type including local, regional and distal. If patients failed in the same breast or chest wall, it was referred as local. If the recurrence were in ipsilateral axilla, internal mammary or supraclavicular region, it was scored as regional. All other sites were called distant failure. We also collected information on site of distant failure including lungs, bones, central nervous system (CNS), liver, non-regional lymph nodes and others.

### Statistical analysis

Fisher’s exact test was used to compare frequencies for categorical variables. The Kaplan–Meier product-limit method was used to determine overall (OS), disease-free (DFS), local failure-free (LFFS), and distant failure-free (DFFS) survival probabilities, with *p*-values for group comparisons based on the log-rank statistic. Adjusted hazard ratios (aHR) and 95 % confidence intervals (CI), were computed using a Cox regression model. Multivariable models were adjusted for age, stage, and grade. Based on the test statistic of Grambsch and Therneau, the parallel (proportional) hazards assumption was not violated in our models. An iterative expectation–maximization procedure was used to account for missing values (imputation efficiency >99 %). Rounding was performed using the method of Holly and Whittemore. Statistical significance was defined as *p* ≤ 0.05. SAS, version 9.4 (Cary, NC) was used for all analyses.

## Results

### Patient and tumor characteristics

Patients were roughly equally distributed between races, with 49 % black (n = 218) and 51 % white (n = 230) (Table 1). More than half (59 %) of patients were between the ages of 41 and 60. Another 18 % were ≤40 years old, and 23 % were ≥61 years old. There was a significant difference in age distribution between black and white participants (*p* = 0.0006), with a greater proportion of white patients being ≥61 years old.

**Table 1:** Patient and tumor characteristics comparison between black and white women

Characteristic	% (n)	Black % (n)	White % (n)	*p* value^†^
Race^a^	100 (448)			
Black		49 (218)		
White			51 (230)	
Age (years)				
≤40	18 (79)	17 (36)	19 (43)	0.0004
41–60	59 (264)	67 (147)	51 (117)	
≥61	23 (105)	16 (35)	30 (70)	
Stage				
I	19 (87)	16 (35)	23 (52)	0.026
II	47 (212)	53 (116)	42 (96)	
III	28 (125)	28 (60)	28 (66)	
IV	5 (24)	3 (7)	7 (17)	
Grade (differentiation)				
1 (Well)	1 (6)	1 (2)	2 (4)	0.79
2 (Moderately)	10 (45)	10 (21)	10 (24)	
3 (Poorly)	89 (397)	89 (195)	88 (202)	

^†^Fisher’s exact test

^a^Non-Hispanic

Of the 448 patients, the largest percentage (47 %, n = 212) presented with Stage II disease followed by stage III disease with 28 % (n = 125). Only 5 % (n = 24) had Stage IV disease at presentation. There was a significant difference in stage of disease between black and white patients (*p* = 0.026), with a greater proportion of white patients having Stage IV disease.

The majority of patients (89 %, n = 397) had Grade 3 disease, as expected for TNBC. There was no significant difference in distribution of disease grade between black and white patients. One hundred forty three (143) patients (32 %) had lymphovascular invasion.

### Treatment

Almost all patients (93 %) received chemotherapy, with 46 % receiving neoadjuvant chemotherapy and 46 % receiving adjuvant chemotherapy (Table 2). Although slightly more black than white patients received neoadjuvant chemotherapy (48 vs 45 %), the percentage patients with a complete response to neoadjuvant chemotherapy was nearly equal between races.

**Table 2:** Treatment characteristics comparison between black and white women

Characteristic	% (n)	Black % (n)	White % (n)	p value^†^
Chemotherapy				
Neoadjuvant	46 (208)	48 (105)	45 (103)	0.055
Complete response	29 (61)	30 (31)	29 (30)	
Partial response	71 (147)	70 (74)	71 (73)	
Adjuvant	46 (208)	48 (104)	45 (104)	
No chemotherapy	7 (32)	4 (9)	10 (23)	
Surgery				
Breast-conserving surgery^a^	45 (203)	53 (116)	38 (87)	0.0010
Mastectomy	47 (211)	38 (83)	56 (128)	
No surgery	8 (34)	9 (19)	7 (15)	
Breast radiation				
Radiation	73 (329)	79 (173)	68 (156)	0.0073
No radiation	27 (119)	21 (45)	32 (74)	

^†^Fisher’s exact test

^a^Lumpectomy

Similarly, 92 % of patients underwent surgery, with 45 % opting for breast-conserving surgery (BCS) and 47 % opting for mastectomy. There was a significant difference in type of surgery between black and white patients, with a greater proportion of black patients having BCS *(p* = 0.0010).

Most patients (73 %, n = 329) underwent radiation therapy. There was a significant difference in utilization of radiation between black and white patients (*p* = 0.0105), with a greater proportion of black patients receiving radiation (*p* = 0.0073).

### Patterns of failure

**Fig. 1:**
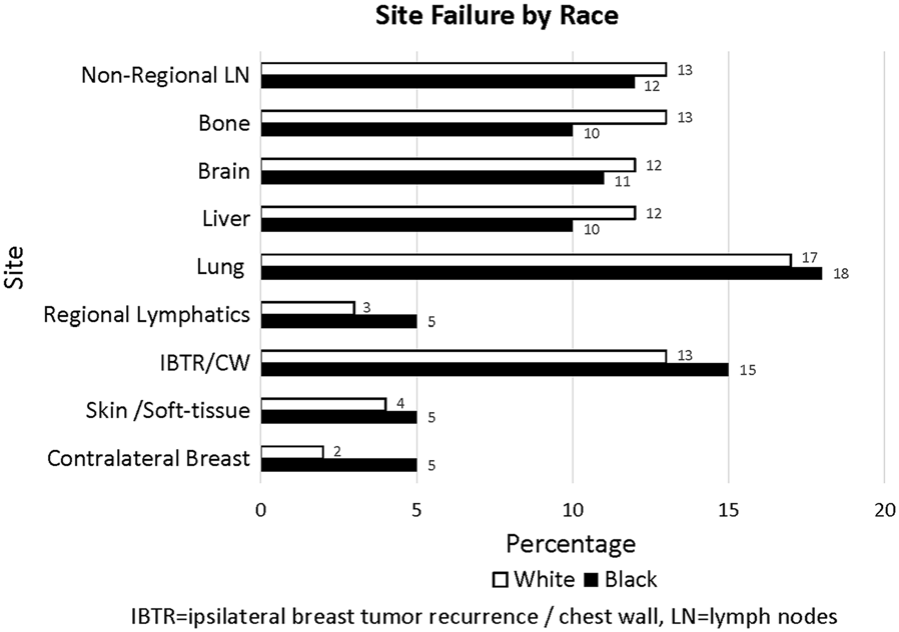
Site failure by race

Lung (18 %) was the most common site of distant failure followed by brain (12 %), liver (11 %) and bone (11 %) (Fig. 1). Approximately 14 % of patients developed local breast or chest wall failure and 3 % of women developed contralateral breast cancer. In addition, we also observed failure involving non-regional lymphatics to be high at 13 %. Only 4 % of patients had skin and soft tissue involvement outside the breast and chest wall. Regional lymph node failure was 4 %. There was no significant difference in sites of overall failure pattern between black and white patients.

### Patterns of survival

**Fig. 2:**
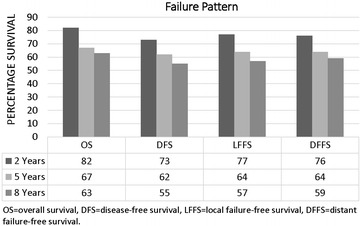
The 2-, 5-, and 8-year overall (OS), disease-free (DFS), local failure-free (LFFS), and distant failure-free (DFFS) survival probabilities

**Fig. 3:**
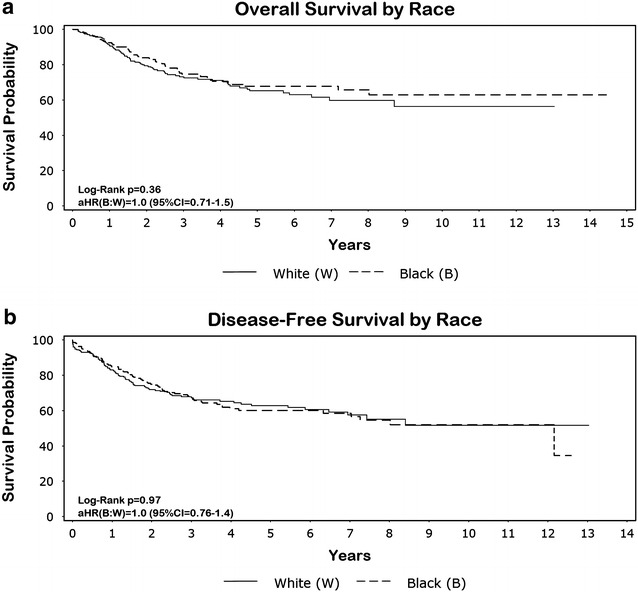
**a**, **b** Overal (OS) and disease-free (DFS) survival by race. Adjusted hazard ratios (aHR) and 95 % confidence intervals (CI) are displayed in the lower left-hand corner of the graph

**Fig. 4:**
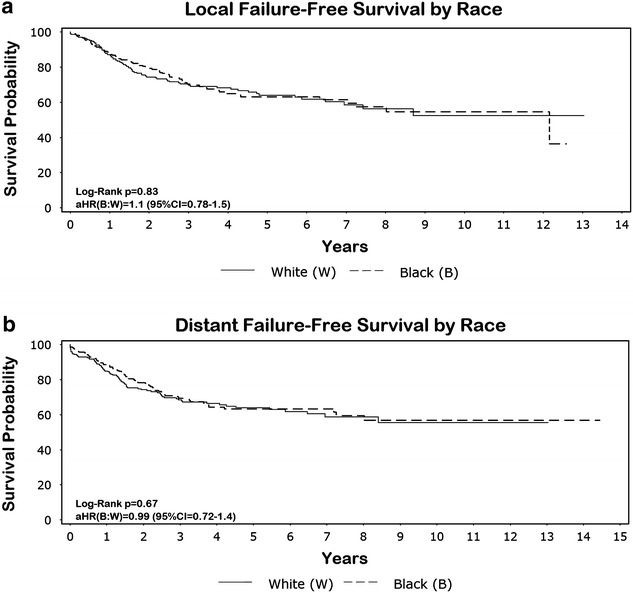
**a, b **Local failure-free (LFFS) and distant failure-free (DFFS) survival by race. Adjusted hazard ratios (aHR) and 95 % confidence intervals (CI) are displayed in the lower left-hand corner of the graph

Follow-up time ranged from 0.14 to 14 years, with the average patient being followed for 3.8 years. Small but continuous occurrences of failure were observed both locally and distally (Fig. 2). Even though there were significant differences seen in age, stage and local treatment distribution between the two groups, survival end points between the two races were similar. For example, the 5-year OS, DFS, LFFS, and DFFS was 68, 60, 63 and 63 % for blacks and 65, 63, 64, and 64 % for whites, respectively. Adjusting for age, stage, and grade, hazard ratio CIs for each survival outcome included unity (Figs. 3a, b and 4a, b). The additional adjustment for year period of diagnosis (≤2000, 2001–2005, ≥2006) or insurance type did not substantively change our findings.

## Discussion

TNBC accounts for only 10–17 % of all breast cancers, yet is responsible for a relatively large proportion of breast cancer deaths owing to its inherent aggressive biology (Dent et al. 2007; Podo et al. 2010). Another aggressive subtype is HER2 positive breast cancer. This type used to have shorter disease-free survival rates until the wide spread availability of HER2 directed therapy utilizing trastazumab with significant improvement in survival in the modern era. Triple negative subtype on the other hand lacks any specific target and currently is subject to vigorous investigations to improve its outcome. TNBC also is a biologically extremely heterogeneous group of breast cancer, with a high likelihood of recurrence during the first 2–3 years after diagnosis (Foulkes et al. 2010). Dent et al. (2007) found that patients with TNBC subtype had more likelihood of distant recurrence and death from breast cancer within 5 years of diagnosis. They also noted that distant recurrence and breast cancer mortality was seen in the first 5–7 years with peak time at 3 years but not after that time period. Liedtke et al. (2008) also reported similar findings. They evaluated response to neoadjuvant chemotherapy among more than 1000 patients from 1985 to 2004 and reported 3 year progression-free survival and OS rates for TNBC were poorer compared with other subtypes. They also reported that death rates were higher only in the first 3 years of diagnosis reaching a plateau after that period. In our study we noted a similar significant drop in OS and DFS in the first 5 years. In addition, we also noted a small but persistent increase in death and recurrence occurring beyond 5 years. This is in contrast with the two previously reported series.

There are conflicting reports about local or loco-regional recurrence rates (LRR) in TNBC following either breast conserving therapy (BCT) or mastectomy. While some reports showed very low incidence of LRR in TNBC of 3–8 % (Freedman et al. 2009; Nguyen et al. 2008; Solin et al. 2009), other series have shown this to be in the range of 10–20 % (Dent et al. 2007; Abdulkarim et al. 2011; Haffty et al. 2006; Voduc et al. 2010). In addition, while several of these reports (Dent et al. 2007; Freedman et al. 2009; Haffty et al. 2006) did not find any difference in LRR between the different subgroups, others have noted increased rates of both local and regional recurrence to be significantly higher in TNBC and HER2 subgroups compared with hormone receptor positive (HR) subtypes (Nguyen et al. 2008; Voduc et al. 2010; Gabos et al. 2010; Wang et al. 2011). Finally, the systemic meta-analysis have suggested a higher rate of local–regional recurrence among TNBC subgroups compared with hormone receptor positive breast cancer either following BCT or mastectomy (Lowery et al. 2012). Our series has a similar 13 % rate of local only recurrence. In addition, we also found a low rate of regional nodal failure of 4 %. While our report did not include other subtypes and therefore cannot be compared among different subgroups, it nonetheless suggests a similar local failure rate compared with other reports. Report by Abdulkarim et al. (2011) and Gabos et al. (2010) suggested that TNBC had inferior local–regional recurrence following mastectomy with/without radiation, but this was not confirmed by others.Thus, there is inadequate information to suggest either BCT or mastectomy is a preferable mode of local treatment. Based on the current information, the decision regarding local–regional treatment should consider disease stage, individual patient’s desire and type of systemic therapy and response.

The metastatic behavior of TNBC also is quite distinct compared with other subtypes. TNBC tends to metastasize more frequently to viscera compared with hormone receptor positive cancers. TNBC has been reported to have lung metastasis as the most frequent site compared with other sites (Anders and Carey 2009; Foulkes et al. 2010; Yagata et al. 2011; Kennecke et al. 2010). It also involves brain/CNS at a relative higher rate and lower rate of bone involvement (Anders and Carey 2009; Foulkes et al. 2010; Yagata et al. 2011; Kennecke et al. 2010) compared with hormone receptor (HR) positive subtypes. Kennecke et al. (2010) reported cumulative incidence of lung metastasis for basal subtype to be 18.5 % followed by 16.6 % of bone, 10.9 % brain, 9.3 % liver and 17.2 % distant nodal failure. Similarly, our report showed the lungs to be the most distant failure site. In addition, we also noted higher incidence of non-regional nodal recurrence. Furthermore, brain, liver and bone involvement were common and in the similar range in our cohort. We did not see a higher rate of brain/CNS metastasis compared with bone involvement as documented in other studies. Since, most of the studies reported relative incidence difference; it is difficult to conclude whether this truly is the case. However, our rate of bone metastasis is similar to the report by Kennecke et al. (2010).

Significant racial disparity is well-known to exist for TNBC incidence. In particularly, black women have a higher likelihood of developing breast cancer with a triple negative phenotype and tend to be younger at diagnosis (Bowen et al. 2008; Huo et al. 2009). Socioeconomics, cultural factors, and lack of adequate screening are believe to be important explanatory factors for this disparity. The issue of race and survival for women with TNBC specific subtypes has conflicting reports. Studies conducted by Bauer et al. (2007b) and Lund et al. (2009) found that black women have a worse survival for TNBC, after controlling for socioeconomic factors, treatment delay and tumor characteristics. Dawood et al. (2009) on the other hand found that the black race was not associated with a detriment in survival or likelihood of attaining a pathological complete remission among patients with TNBC.

Our cohort of TNBC patients is unique in that there is an almost equal representation of black and white women, with a large sample size for each race. We found that among TNBC patients, there was no statistical difference in all 4 survival measures between black and white women. This is interesting, given that in our study white patients were more likely to have Stage IV disease and blacks were more likely to undergo BCS and RT. We also did not find any significant difference in pattern of failure between the two races. Future study is needed to determine if our findings reflect a unique regional variation for TNBC (DeSantis et al. 2016).

Our study has several limitations including its observational design and paucity of information on post-surgery radiation dose and field employment. Information on neighborhood socioeconomic status, racial residential segregation, education level, and utilization/access to long term follow-up care also was not available for our analysis. Furthermore, the adjustment variables used in our multivariable model may have been incomplete (e.g., differences in tumor severity even within the categories we used for adjustment). However, our study is a large dataset including only triple negative subtype, with an equal representation of black race. This uniquely allowed us to show significant differences in demography and treatment but no differences in OS, DFS, LFFS, or DFFS. The lack of targeted hormonal therapy for TNBC coupled with the aggressive nature of this tumor type may supersede the influence of race. Nonetheless, the factors affecting failure patterns and survival outcomes have a complex relationship and future research is needed to better understand our results.

## Conclusion

This is a large TNBC data set showing demographic treatment comparisons between black and white patients with no difference in failure patterns and survival outcomes. For both races, distant failure was the predominant pattern of failure with lung being the most common site. Non-regional distant nodal failure was higher compared with regional nodal failure.
